# Roxadustat: Not just for anemia

**DOI:** 10.3389/fphar.2022.971795

**Published:** 2022-08-29

**Authors:** Xiaoyu Zhu, Lili Jiang, Xuejiao Wei, Mengtuan Long, Yujun Du

**Affiliations:** ^1^ Department of Nephrology, The First Hospital of Jilin University, Changchun, Jilin, China; ^2^ Physical Examination Center, The First Hospital of Jilin University, Changchun, Jilin, China

**Keywords:** roxadustat, hypoxia-inducible factor, renal anemia, fibrosis, metabolism, inflammation, oxidative stress, angiogenesis

## Abstract

Roxadustat is a recently approved hypoxia-inducible factor prolyl hydroxylase inhibitor that has demonstrated favorable safety and efficacy in the treatment of renal anemia. Recent studies found it also has potential for the treatment of other hypoxia-related diseases. Although clinical studies have not yet found significant adverse or off-target effects of roxadustat, clinicians must be vigilant about these possible effects. Hypoxia-inducible factor regulates the expression of many genes and physiological processes in response to a decreased level of oxygen, but its role in the pathogenesis of different diseases is complex and controversial. In addition to increasing the expression of hypoxia-inducible factor, roxadustat also has some effects that may be HIF-independent, indicating some potential off-target effects. This article reviews the pharmacological characteristics of roxadustat, its current status in the treatment of renal anemia, and its possible effects on other pathological mechanisms.

## Introduction

Hypoxia-inducible factor (HIF) is a key regulator of the body’s response to hypoxia. This protein is a heterodimer that has an oxygen-sensitive *a* subunit and a constitutively expressed *ß* subunit. The prolyl hydroxylase domain (PHD) is an oxygen sensitive enzyme with three subtypes (PHD1, PHD2, and PHD3) that regulates the activity of HIF-α (Beuck et al., 2012; Myllyharju, 2013). Under normoxic conditions, PHD catalyzes the degradation of the HIF-α subunit, leading to its inactivation. However, hypoxic conditions inhibit PHD activity, leading to stabilization and accumulation of HIF-α in the cytoplasm (Fandrey, 2004); under these conditions, HIF-α translocates into the nucleus, dimerizes with HIF-β, and activates the transcription of many genes (Akizawa et al., 2020a). In 1992, Wang et al. (1995) first discovered that HIF binds to the hypoxia response element (HRE) in the enhancer region of the erythropoietin gene (*EPO*), and was responsible for the induction of *EPO* and other oxygen-sensitive genes during hypoxia. To date, three distinct HIF-α subtypes have been identified: HIF-1α, HIF-2α, and HIF-3α. HIF-1α is primarily regulated by PHD2 (Cioffi et al., 2003), is expressed in almost all cell types, and activates the transcription of many genes, including those that function in iron metabolism, angiogenesis, energy metabolism, mitochondrial metabolism, inflammation, cellulose production, and cell fate (Semenza, 2012; Locatelli et al., 2017). HIF-2α is mainly regulated by PHD1 and PHD3 (Appelhoff et al., 2004; Joharapurkar et al., 2018), is more restricted to specific cell types, such as renal interstitial fibroblast-like cells and endothelial cells, and is a major transcription factor regulating *EPO* expression and iron transport (Haase, 2013). The function of HIF-3α remains uncertain, although some research showed it down-regulated the expression of the other two HIF genes (Hara et al., 2001).

**FIGURE 1:**
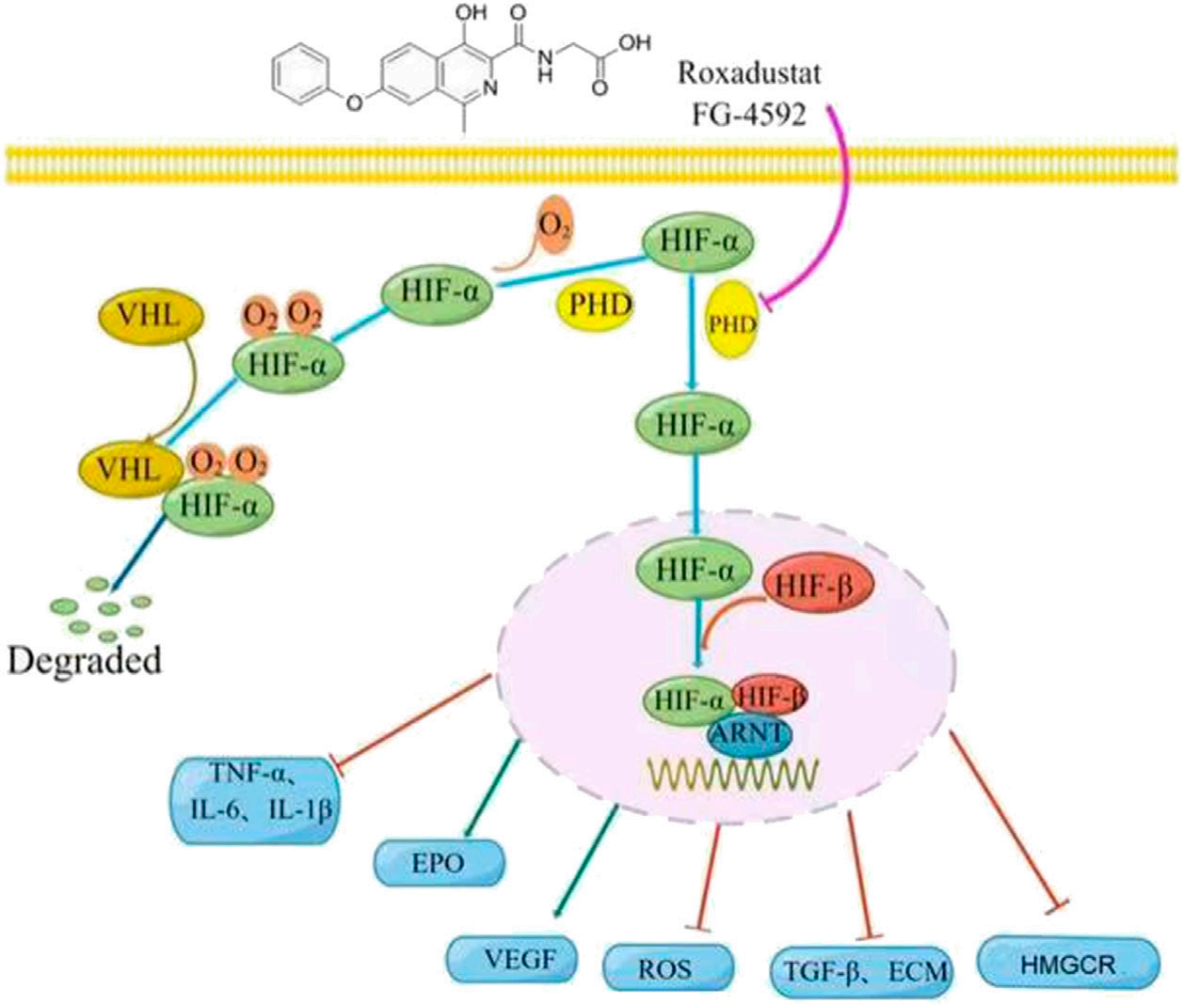
Roxadustat mechanism of action. Left: Under normoxic conditions, the prolyl hydroxylase domain (PHD) hydroxylates the oxygen-sensitive *α* subunit of hypoxia-inducible factor (HIF-α), and the von Hippel-Lindau tumor suppressor (VHL) recognizes the hydroxylated HIF-α, which is then ubiquitinated and degraded by ubiquitin. Right: Roxadustat (or hypoxia) inhibits the activity of PHD, leading to the accumulation of HIF-α, which subsequently moves into the nucleus where it forms a transcriptional complex with HIF-β and aryl hydrocarbon receptor nuclear translocator (ARNT), and then binds to the hypoxia response element, thereby regulating the expression of many genes (TNF-α, IL -β, IL-6, EPO, VEGF, TGF-β, HMGCR, etc.), as well as the production of ROS and ECM.

Roxadustat (FG-4592) is an orally bioavailable and reversible hypoxia-inducible factor prolyl hydroxylase inhibitor (HIF-PHI) that inhibits PHD by mimicking 2-oxoglutarate, one of its substrates (Akizawa et al., 2019). This drug stabilizes the level of HIF and stimulates erythropoiesis in a dose-dependent manner (Chang et al., 2019). Clinical trials of roxadustat began in November 2005 (Grzeszczak et al., 2021) and it was first approved in China for treatment of anemia patients receiving hemodialysis or peritoneal dialysis in December 2018 (Dhillon, 2019). In August 2019, roxadustat was approved for the treatment of anemia in Chinese patients with chronic kidney disease (CKD) but not receiving dialysis (Li et al., 2020a). Roxadustat has received increasing attention from researchers and nephrologists in various disciplines since its approval. Studies have shown that roxadustat has similar effects on the three subtypes of proline hydroxylase (PHD1, PHD2, and PHD3) (Jatho et al., 2022). In addition to the treatment of renal anemia, roxadustat may also protect against other hypoxia-related diseases, including chronic inflammation, fibrosis, ischemia, and even cancer. In this review, we summarize the pharmacological characteristics of roxadustat and describe recent research that examined its potential for use in pathologies other than renal anemia in an effort to provide an objective, in-depth, and up-to-date understanding of this medication (Figure 1).

## Pharmacological characteristics of roxadustat

Roxadustat is an oral, potent, and reversible small molecule HIF-PHI whose molecular weight is 352.34 g/mol and chemical formula is C_19_H_16_N_2_O_5_ (Czock and Keller, 2022). It is a second-generation HIF-PHI that was synthesized by adding a phenoxy group to carbon-7 of the quinoline core of its precursor, FG-2261 (Del Vecchio and Locatelli, 2018). Roxadustat has a good pharmacokinetic profile, its solubility depends on pH (Shibata et al., 2018), and it is rapidly absorbed in healthy subjects and patients with moderate hepatic impairment. After oral administration, it usually reaches a maximum blood concentration in about 2 h (Groenendaal-van de Meent et al., 2016a). The absorption of roxadustat is independent of food (Shibata et al., 2019), and body weight, age, gender, race, and drug dose have no significant effects on its pharmacokinetics (Czock and Keller, 2022). After oral absorption, roxadustat is transported to the liver mainly *via* phase I oxidation by cytochrome P450 2C8 and phase II glucuronidation by uridine diphosphate glucuronyltransferase (UGT1A9) (Groenendaal-van de Meent et al., 2021a). Its elimination half-life is approximately 9.6–16 h in healthy volunteers and approximately 18 h in patients with impaired renal function (Rekić et al., 2021; Czock and Keller, 2022). Roxadustat is a lipophilic acid, and is therefore tightly bound (approximately 99%) to plasma proteins, and cannot be significantly removed by dialysis (Provenzano et al., 2020). Because roxadustat is mainly metabolized in the liver, the European Drug Administration suggested the dose should be reduced to half when initially given to patients with moderate liver cirrhosis (Child Pugh B), and that patients with severe liver cirrhosis (Child Pugh C) should not receive roxadustat (Czock and Keller, 2022).

Patients with CKD typically need to take other drugs to treat the many complications of this disease, and drug interactions should be considered when treating these patients. Lanthanum carbonate, carbon adsorbents, omeprazole, and warfarin have no clinically relevant effects on the pharmacokinetics of roxadustat (Groenendaal-van de Meent et al., 2016b; Groenendaal-van de Meent et al., 2018; Shibata et al., 2018; Shibata et al., 2019). However, phosphorus-lowering drugs, such as calcium acetate and sevelamer, may form insoluble chelates with roxadustat and reduce its absorption (Groenendaal-van de Meent et al., 2021b). In addition, roxadustat significantly increases the blood level of statins because it inhibits the pharmacokinetic interaction of organic anion transporting polypeptide 1B1/B3 (OATP1B1/B3) with statins (Groenendaal-van de Meent et al., 2022). Therefore, when statins are co-administered with roxadustat, adverse reactions should be evaluated and the statin dose should be reduced or the interval between the roxadustat doses should be increased. Roxadustat is usually administered 2 to 3 times per week for treatment of anemia in CKD patients, and the plasma levels generally return to very low levels between doses, without significant drug accumulation (Provenzano et al., 2020). Current clinical studies support its efficacy and safety, but more in-depth and long-term studies are needed, especially before its indications can be expanded.

## Effect of roxadustat on anemia

Anemia often accompanies CKD, and is associated with a significantly increased risk of morbidity, mortality, and cardiovascular events (Chen et al., 2017). Previous studies showed that the prevalence of anemia in patients with CKD (15.4%) is twice that of the general population (7.6%), and the prevalence increases as CKD progresses (Stauffer and Fan, 2014). Thus, in the United States, the prevalence of anemia is 8.4% in patients with CKD stage 1 and is 53.4% in patients with CKD stage 5 (Akizawa et al., 2020b). In China, more than 90% of the 500,000 patients undergoing dialysis have anemia (Nakhoul and Simon, 2016). The current treatments for anemia and CKD are generally erythropoietin stimulating agents (ESAs), iron supplements, and blood transfusions. When an ESA is used to treat renal anemia, it can increase the hemoglobin (Hb) level, reduce the need for blood transfusions, and improve quality-of-life, and these drugs have therefore been a key treatment for anemia in patients with CKD since 1989 (Kaplan, 2019). However, treatment with ESAs, especially when aiming to achieve normal levels of Hb, may increase the risk of cardiovascular disease (CVD), myocardial infarction, hypertension, and thromboembolism (Singh et al., 2006; Zhang et al., 2021a). Another disadvantage of ESAs is that they are administered intravenously or subcutaneously. Iron therapy may also require injections and hospital visits, and red blood cell transfusions significantly increase the cost of anemia treatment and may lead to infection, allosensitization, and rejection of kidney transplants. All of these factors can affect treatment compliance and outcome in non-hemodialysis and peritoneal dialysis patients who have anemia (Besarab et al., 2015; Abdelazeem et al., 2021). In addition, approximately 10% of patients are resistant to ESAs and require higher doses to achieve the recommended Hb target (Akizawa et al., 2020c). However, use of high-dose ESAs appears to increase the risk of adverse cardiovascular effects and mortality (Besarab et al., 2015; Barratt et al., 2021). These findings led the US Food and Drug Administration (FDA) to recommend use of the lowest possible dose of an ESA that can achieve an adequate Hb level without the need for transfusions (Becker and Saad, 2017). Although renal anemia is a serious and widespread problem, many patients with CKD still suffer from anemia due to inadequate treatment. Therefore, a safer and more effective method for treatment of anemia in CKD patients is urgently needed.

Three researchers who described the mechanism of HIF in response to hypoxia received the Nobel Prize in 2019 (Wilson et al., 2020). Roxadustat, as a novel oral HIF-PHI used for treatment of renal anemia, can regulate many of the pathological responses related to renal anemia. In particular, an intermittent dosing regimen of roxadustat (2 or 3 times per week) induces a transient elevation in endogenous EPO to near the normal physiological range, but below the level achieved by an intravenous ESA (Besarab et al., 2016). Roxadustat can also increase Hb to a level similar to that achieved by an ESA without substantially increasing the risk of cardiovascular events (Liu et al., 2020). Another consideration when using an ESA is that iron is required for erythropoiesis (Chen et al., 2019a), and hepcidin (a key factor regulating iron homeostasis) increases with inflammation and limits intestinal absorption of iron, potentially causing ESA resistance (Akizawa et al., 2020b; Zhang et al., 2021b). Inflammation suppresses the response to an ESA, but the effects of roxadustat on Hb appear to be unaffected by inflammation (Bradbury et al., 2009; Akizawa et al., 2020d). Compared with ESAs, roxadustat appears to cause greater suppression of hepcidin (Ogawa et al., 2020; Grzeszczak et al., 2021). As a likely consequence, roxadustat is effective in some individuals who are relatively resistant to ESAs. Consistent with this view, there is evidence that roxadustat prevents ESA-hyporesponsiveness and anemia in patients with myelodysplastic syndrome (Yu et al., 2020; Parisi et al., 2021). In addition, Li et al. (2021a) showed that roxadustat significantly increased the Hb level in patients with renal transplantation anemia without affecting renal function or increasing rejection. In contrast to ESAs, roxadustat is an oral medication, it can be stored at room temperature, and it improves the efficacy of oral iron and reduces the need for intravenous iron. These are clear advantages for patients with CKD, especially those who have anemia and are non-dialysis dependent (NDD) or using peritoneal dialysis.

Many studies have reported the efficacy and safety of roxadustat when used as a treatment for renal anemia. However, there is some evidence that roxadustat can cause a variety of side effects, mainly diarrhea, vomiting, peripheral edema, headache, back pain, fatigue, and hyperkalemia (Akizawa et al., 2020c; Liu et al., 2020). In addition, HIF functions in the progression of polycystic kidney disease (PKD), and roxadustat may promote the growth of renal cysts (Liu et al., 2021). A case report found that roxadustat reduced the TSH levels in hemodialysis patients, but the mechanism and clinical significance of this effect is unclear (Tokuyama et al., 2021). Another consideration is that different countries have approved different initial doses of roxadustat, and there is also no unified clinical guideline regarding the starting and ending times of treatment. A document released by the U.S. FDA on its official website on 15 July 2021 questioned the cardiovascular safety of roxadustat. In particular, for dialysis dependent (DD) patients, roxadustat had a higher cardiovascular risk than epoetin alfa; for NDD patients, roxadustat had a higher cardiovascular risk than placebo. Therefore, the FDA refused to approve the listing of roxadustat in the United States. Therefore, large, high-quality multinational studies with long-term evaluations are needed to further examine different dosing strategies. In addition to roxadustat, other HIF-PHIs have been used clinically or are undergoing clinical trials for the treatment of CKD-related anemia (Beuck et al., 2012; Dhillon, 2020; Markham, 2020; Figg et al., 2021; Markham, 2021; Dhillon, 2022). Although all HIF-PHIs have similar mechanisms of action, their specific effects on cells and preferred targets may differ. This paper summarizes recent research on roxadustat and other HIF-PHIs, and describes their half-lives, targets, indications, and major adverse effects (Table 1).

**TABLE 1:** The PHD inhibitors have been trialled in Humans.

Molecule	Half life	Main target	Indications	Major adverse reactions	Sponsor	Investigatinal status
Roxadustat	9.6–12 h	PHD1-3	Dialysis and non-dialysis; CKD anemia	Diarrhea, vomiting, peripheral edema, headache, back pain, fatigue, and hyperkalemia	Fibrogen	It is described as a second-generation HIF-PHI, and listed in China in December, 2018 (Czock and Keller, 2022)
FG-2216	14 h	PHD1-3	Anemia including renal anemia, sickle cellemia, chemotherapy-induced anemia	In 2007, a case of death due to fulminant hepatitis occurred in the course of a phase II study. (The adverse event was later declared to be unrelated to the drug, but no further clinical studies have been reported for FG-2216)	Fibrogen	Phase II (Beuck et al., 2012)
Daprodustat	4 h	PHD1 and PDH3 are preferred	Chronic renal anemia	Retinal hemorrhage and anaphylaxis, hypertension, increased myocardial infarction and heart failure deterioration events, and showed tumor effects in patients with NDD	GlaxoSmithKline	Listed in Japan in June 2020 (Dhillon, 2020)
Vadadustat	4.5 h	PHD3	Dialysis and non-dialysis; CKD anemia	Gastrointestinal reaction, hypertension, hyperkalemia	Akebia therapeutics	Listed in Japan in June 2020 (Markham, 2020)
Enarodustat	1.4 h	PHD1-3	Chronic renal anemia	Respiratory tract infection, gastrointestinal reaction	Japan Tobacco Inc.	Listed in Japan in September 2020 (Markham, 2021)
Desidustat	6.9–13 h	PHD1-3	Dialysis and non-dialysis; CKD anemia	Dizziness, respiratory infections, and gastrointestinal reactions	Zydus Cadila	Phase III (Dhillon, 2022)
Molidustat	7–13 h	PHD1-3, especially PHD3	Chronic kidney disease or end-stage; nephropathy-associated anemia	Increased levels of inflammatory markers CRP, infections and gastrointestinal reactions	Bayer health care	Listed in Japan in January 2021 (Figg et al., 2021)

**FIGURE 2:**
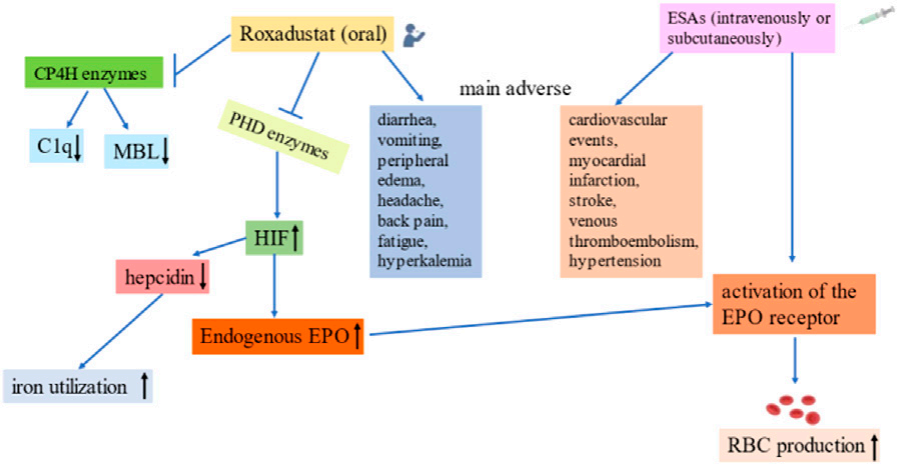
Comparison of roxadustat and erythropoietin stimulating agents. Far-left: Possible off-target effects of roxadustat. Left: Roxadustat promotes endogenous EPO production, increases EPO receptor activity, reduces the hepcidin level, and increases iron utilization. Middle-Left: Common adverse effects of roxadustat. Middle-Right: Common adverse effects of ESAs. Far-Right: Effects of erythropoietin stimulating agents on anemia.

In addition, considering the breadth of biological processes regulated by HIF, roxadustat may have clinical effects beyond the stimulation of erythropoiesis. Another important consideration is that roxadustat was designed as an analogue of 2-oxoglutarate (2-OG) that inhibits 2-OG-dependent dioxygenase (2-OGDD). This enzyme is in a superfamily whose members have a wide range of biological functions. Thus, PHD enzymes may not be the only substrates of roxadustat, and roxadustat (or similar PHD enzyme inhibitors) could possibly inhibit other 2-OGDDs, which may lead to “off-target” effects beyond its effects on HIF and PHD (Vissers et al., 2014). For example, collagen prolyl 4-hydroxylase (CP4H) is a type of non-heme iron (II)-containing 2-OGDD (Vasta and Raines, 2016). The PHD and CP4H enzymes are both proline-4-hydroxylases. Roxadustat and CP4H inhibitors are structural analogues, and recent studies found that roxadustat can inhibit the hydroxylation and secretion of complement C1q by acting on CP4H. This suggests that prolonged use of roxadustat for treatment of renal anemia may eventually decrease the level of C1q, which may have detrimental clinical effects (Kiriakidis et al., 2017). Mannose-binding lectin (MBL), a serum C-type lectin produced by the liver, plays an important role in the innate immune response by activating the lectin pathway of the complement system and subsequent inflammatory mechanisms (Wu et al., 2020). CP4H mediates the hydroxylation of MBL, and after multiple post-translational modifications, MBL oligomerizes to a high molar weight (HMW) form. Bhute et al. (2020) found that roxadustat inhibited the proline hydroxylation of MBL and the formation of HMW oligomers by inhibiting the activity of CP4H. This may be considered an off-target effect of certain PHD enzyme inhibitors, including roxadustat, and these effects may be beneficial, neutral, or harmful. Figure 2 compares the effects of roxadustat and ESAs when used for treatment of anemia.

## Effect of roxadustat on fibrosis

Tissue fibrosis underlies most chronic diseases that have high levels of morbidity and mortality, but treatment of fibrosis remains challenging. Hypoxia is an important microenvironmental factor that promotes the development of tissue fibrosis, and HIF is a major regulator of cellular adaptation to ischemia and hypoxia. Numerous studies confirmed an association of HIF with fibrosis. For example, Kapitsinou et al. (2012) found that stabilization of HIF prior to the onset of ischemia reduced renal fibrosis and prevented anemia. Yu et al. (2012) studied rats with subtotal nephrectomy and found transient activation of HIF-α in the residual kidney during the early postoperative period, and activation of HIF-α during a later stage that reduced tubulointerstitial fibrosis. This suggests that appropriate short-term increases in the level of HIF-α may prevent renal fibrosis. Recently, Wu et al. (2021) studied a unilateral renal ischemia-reperfusion injury (IRI) model and reported that roxadustat significantly attenuated renal fibrosis, and that the increase in HIF may have provided this protective effect by maintaining renal function and preventing the transition from AKI to CKD. Huang et al. (2020) showed that roxadustat inhibited experimental pulmonary fibrosis by regulating the TGF-β1/Smads signaling pathway *in vitro* and *in vivo*. Li et al. (2021b) studied a folic acid (FA)-induced AKI model and found that roxadustat pretreatment promoted the regeneration of renal tubular structures 7 days after FA injection. They also found that roxadustat inhibited ferroptosis and inflammation due to activation of Nrf2 by Akt/GSK-3β, thereby inhibiting interstitial fibrosis (Li et al., 2020b).

However, the role of HIF in fibrosis is still controversial, and there is evidence that hyperactivation and prolonged exposure to HIF may actually increase renal fibrosis. For example, Kimura et al. (2008) studied a 5/6 nephrectomy model (removal of one kidney and 2/3 of the other kidney) and found that VHL deletion led to increased HIF-1α expression and increased renal fibrosis, and that the application of anti-HIF-1α drugs inhibited the progression of renal fibrosis in the unilateral ureteral obstruction (UUO) mouse model. Higgins et al. (2007) showed that HIF-1α enhanced the epithelial-mesenchymal transition (EMT) *in vitro*, and that increased expression of HIF-1α was associated with tubulointerstitial damage in patients with CKD. These authors also demonstrated that genetic ablation of HIF-1α from epithelial cells inhibited the development of tubulointerstitial fibrosis in the UUO model. Studies of pulmonary fibrosis reported that HIF-1α activation induced cell proliferation, adhesion, and secretion of extracellular matrix (ECM), and these led to lung inflammation and fibrosis under hypoxic conditions (Huang et al., 2020).

**FIGURE 3:**
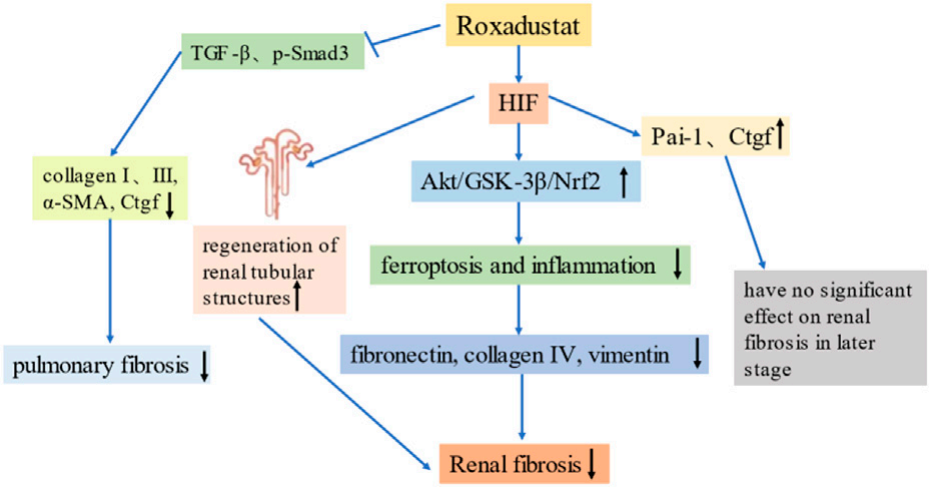
Roxadustat reduces fibrosis. Far-left: Roxadustat inhibits the expression of TGF-β and p-Smad3, and reduces the levels of collagen, α-SMA and Ctgf, thereby alleviating pulmonary fibrosis. Left: Roxadustat upregulates HIF, promotes the regeneration of renal tubular structures, and alleviates renal fibrosis. Middle: Roxadustat upregulates HIF, promotes the Akt/GSK-3β/Nrf2 pathway, reduces ferroptosis and inflammation, and thereby reduces the production of fibrotic proteins and renal fibrosis. Right: Roxadustat upregulates HIF, which may upregulate the expression of pro-fibrotic genes (Pai-1 and Ctgf), but does not significantly promote renal fibrosis in the later stages of disease.

The role of HIF in promoting the progression of fibrosis appears contrary to its organ-protective role during early kidney injury. These different effects may be explained by differences in the timing of administration and the extent and duration of HIF activation. Activating HIF at an appropriate level and within a well-regulated range may promote cellular adaptation to hypoxia and ischemia, and therefore play a protective role. Kabei et al. (2020) simulated renal fibrosis in the UUO model and found that a high dose of roxadustat (50 mg/kg/day) significantly increased the expression of pro-fibrotic genes after 3 days, but there was no effect after 7 days. The effect of roxadustat on renal fibrosis may also be dose- and time-dependent, in that it may promote renal fibrosis during the early stage of disease, but this pro-fibrotic effect gradually declines, and there is a protective effect during a later stage (Kabei et al., 2018). To date, no clinical trials have reported significant adverse events or significant off-target effects. However, given the role of HIF and roxadustat in fibrosis, it is necessary to consider possible adverse effects by carefully considering the dose and timing of treatment. Figure 3 summarizes the mechanisms by which roxadustat reduces fibrosis.

## Effect of roxadustat on lipid metabolism

Clinical research showed that roxadustat significantly reduced the level of plasma total cholesterol in patients with CKD due to an increased HIF-mediated degradation of 3-hydroxy-3-methylglutaryl (HMG) -CoA reductase, and that this effect occurred regardless of statin use (Chen et al., 2019a). In addition, roxadustat lowered the level of LDL-cholesterol and triglycerides, and increased the ratio of HDL-cholesterol to LDL-cholesterol, and the cholesterol level returned to the pre-treatment level after discontinuation of roxadustat (Del Vecchio and Locatelli, 2018; Sanghani and Haase, 2019). These are important findings, because reductions in the levels of total cholesterol, LDL-cholesterol, and triglycerides reduce cardiovascular morbidity and mortality (Kassimatis and Goldsmith, 2014). Therefore, the effects of roxadustat on lipid metabolism may reduce the risk of CKD and CVD. Mi et al. (2020) found that roxadustat reduced the abnormal accumulation of lipids in cells and zebrafish that had a deficient *ATP7B* gene. Hasegawa et al. (2020) showed that HIF stabilizers counteracted the altered renal energy metabolism that occurs during diabetic nephropathy by downregulating fatty acid and amino acid metabolism and upregulating glycolysis, effects may inhibit the progression of diabetic kidney disease (DKD).

However, Li et al. (2021c) showed that roxadustat reduced fatty acid oxidation in renal tubular epithelial cells, and also inhibited lipid metabolism and caused significant lipid accumulation, alterations that may contribute to renal pathology. Kaplan (2020) suggested that roxadustat promoted the switch from aerobic metabolism to glycolysis by increasing the level of HIF-1α. They concluded that this allowed cells to adapt to hypoxia and reduced the overproduction of mitochondrial reactive oxygen species (ROS), but it may also have increased the accumulation of lipids and lactic acid, thus increasing the risk of renal fibrosis. Acidosis caused by the overproduction of lactic acid leads to the release of intracellular potassium ions, and this may explain the presence of hyperkalemia in some patients taking roxadustat. Thus, roxadustat appears to have complex and diverse effects on lipid metabolism. The effect of roxadustat-mediated alterations of lipid metabolism on patient outcome is a topic that requires further research.

## Effect of roxadustat on inflammation

Inflammation is a defense response, but persistent and excessive inflammation occurs in many acute and chronic diseases. Previous studies showed that HIF had anti-inflammatory effects and promoted the resolution of inflammation (Kiers et al., 2016). Cheng et al. (2019) found that early intermittent hypoxia preconditioning of rat skeletal muscle inhibited inflammation and had a protective effect on acute IRI of skeletal muscle. In addition, upregulation of HIF-2α can reduce inflammasome hyperactivation and prevent cell death (Li et al., 2021d). Other research found that roxadustat corrected the inflammatory anemia that was induced in an animal model by peptidoglycan polysaccharide (PG-PS) (Del Balzo et al., 2020). Yang et al. (2018) studied an animal model of cisplatin-induced AKI and showed that roxadustat significantly reduced the levels of TNF-α, IL-1β, IL-6, and other inflammatory cytokines due to its effect on HIF. Han et al. (2020) demonstrated that roxadustat significantly reduced inflammation *in vivo* and *in vitro*, and reduced acute lung injury in mice caused by sepsis due to its upregulation of *HIF-1α* and heme oxygenase 1 (*HO-1*). Another study of a mouse model of dextran sulfate sodium (DSS)-induced colitis showed that activation of HIF-1 had a protective effect, and suggested that PHIs have potential as a novel treatment for inflammatory bowel disease (Cummins et al., 2008).

However, there is currently no consensus regarding the anti-inflammatory function of HIF. Previous studies reported a bidirectional interaction between inflammation and hypoxia (Kerber et al., 2020). In particular, hypoxia induces and promotes inflammation in a variety of pathological conditions, and inflammatory lesions can also lead to the formation of a severe hypoxic microenvironment. A study that examined an animal model of inflammatory bowel disease reported that the diseased tissue and gut had upregulation of HIF-1α and hypoxia (Karhausen et al., 2004). Inflammation promotes cellular hypoxia, and IL-1β increases hypoxia and the levels of HIF-1α in human breast cancer cell lines (Naldini et al., 2010). Yamaguchi et al. (2015) studied the CCAAT/enhancer-binding protein delta (CEBPD), which functions in inflammatory responses, and showed that inflammatory cytokines induced the expression of CEBPD, increased the level of HIF-1α, and promoted tubulointerstitial inflammation. There are also disparate findings regarding the regulation of inflammation by roxadustat. Some evidence indicates that roxadustat inhibits cellular and humoral immunity in mixed lymphocyte reactions (Eleftheriadis et al., 2020). In addition, clinical studies of roxadustat found it was effective in the treatment of renal anemia, but that the incidence of urinary tract infections and pneumonia appeared to be higher in a roxadustat group than a placebo group, suggesting that roxadustat may lead to immunosuppression (Chen et al., 2019b; Akizawa et al., 2020a). Because inflammation underlies so many acute and chronic diseases, more research is needed to clarify the effect of roxadustat on this complex physiological response.

## Effect of roxadustat on oxidative stress

Oxidative stress occurs when there is an imbalance between oxidative and antioxidative reactions (Podkowińska and Formanowicz, 2020), and ROS are responsible for most oxidative stress in biological systems. ROS are mainly derived from the electron transport chain, endoplasmic reticulum, and reduced nicotinamide dinucleotide phosphate (NADPH) oxidases (NOXs) in the mitochondria, and the production and clearance of ROS are tightly controlled by the antioxidant system (Aranda-Rivera et al., 2021). When ROS are at normal physiological concentrations, they can also function as second messengers that induce the activation of various signaling pathways, so an appropriate level of ROS is crucial for allowing cells to engage in normal functions (Lee et al., 2019). However, mitochondrial dysfunction and upregulation of NOXs can cause excessive ROS production and irreversible damage of cells. Therefore, tight regulation of the redox balance is necessary to keep ROS at a suitable concentration.

HIF functions as a key nuclear transcription factor that maintains redox function and oxygen homeostasis (Wang et al., 1995). HIF-1α inhibits ROS formation by inducing the expression of genes that function in antioxidant defense (Li et al., 2018). Furthermore, upregulation of HIF-1α shifts metabolism from oxidative phosphorylation to glycolysis, and this can change the redox potential and reduce mitochondrial generation of ROS (Li et al., 2021c). For example, a study of hepatocellular carcinoma (HepG2) cells reported that HIF-1α upregulated the thioredoxin (TXN), a common anti-oxidant, and thereby reduced oxidative stress (Zhao et al., 2015). However, HIF has broad effects, and the relationship between oxidative stress and HIF-1 may be far more complex. In particular, there may be a positive feedback loop between ROS and HIF-1α, as indicated by a study which showed that an increased level of HIF-1α contributed to the formation of ROS, especially mitochondrial ROS (Agani et al., 2000). There is also evidence that overproduction of ROS during hypoxia increased the activity of HIF-1α *via* the phosphorylation of PI3K/AKT and ERK (Koshikawa et al., 2009). Excess ROS can also activate the NF/κB and TGF-β signaling pathways, thereby promoting the expression of HIF-1α (Zhang et al., 2021c). In contrast, ROS can reduce the level of HIF-1α by upregulating PHD2 or supplying oxygen under hypoxic conditions (Chen et al., 2018).

**FIGURE 4:**
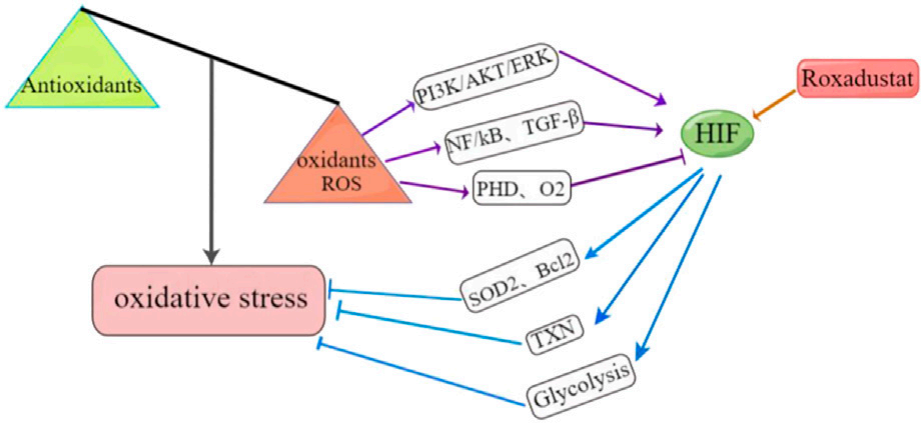
Roxadustat reduces oxidative stress by upregulating HIF. Left: Oxidative stress occurs when there is an imbalance between oxidative and antioxidative reactions. Bottom-Right: Roxadustat upregulates HIF, which reduces oxidative stress by upregulating two target genes (SOD2, Bcl2), glycolysis, and thioredoxin (TXN). Top-Right: ROS overproduction also increases HIF activity by upregulating the PI3K/AKT/ERK pathway, activates the NF/kB and TGF-β signaling pathways, and promotes the expression of HIF. Conversely, ROS can also reduce the HIF level by upregulating PHD enzymes or supplying excess oxygen.

Although current findings suggest the relationship of ROS and HIF is complex, and even differs in different cells and tissues, no clinical studies of roxadustat have reported elevated ROS levels. In fact, there is evidence that roxadustat reduced ROS production and attenuated FA-induced kidney damage (Li et al., 2021b). IRI is the most common cause of acute kidney injury. In particular, during the reperfusion period, abundant ROS are generated due to restoration of the oxygen supply, and this causes cell damage. Eleftheriadis et al. (2021) showed that roxadustat increased the levels of HIF-1α and LDHA in reoxidized cells, but did not affect ROS production. Chen et al. (2021) studied the *in vitro* effects of roxadustat as a treatment for bone fracture and showed that silencing of HIF-1α increased the intracellular concentration of ROS and decreased the survival rate of bone marrow mesenchymal stem cells. Roxadustat also promoted the proliferation and migration of bone marrow mesenchymal stem cells by eliminating ROS, and accelerated fracture healing. Long et al. (2020) studied doxorubicin-induced cardiotoxicity and found that roxadustat reduced oxidative stress in damaged cardiomyocytes by upregulating the expression of HIF-1α and two target genes (*SOD2* and *Bcl-2*), and this ultimately inhibited cardiomyocyte apoptosis and protected cardiac function. Considering that HIF functions in complex networks that are related to the onset and progression of many diseases, and the need to assure safety when administering roxadustat and other drugs, more studies are needed to examine the role of HIF in the pathology of different diseases, and to examine the possible adverse effects of roxadustat treatment. Figure 4 summarizes the mechanisms by which roxadustat reduces oxidative stress.

## Effect of roxadustat on angiogenesis

Blood supplies oxygen and nutrients to cells and tissues, and abnormalities in vascular structure and angiogenesis that lead vascular leakage and reduced blood flow contribute to the pathologies of many disease processes (Liu et al., 2018; Nishide et al., 2020). Therefore, the restoration of vascular function and normalization of angiogenesis may be effective strategies for treatment of different diseases. The main mediator of angiogenesis is vascular endothelial growth factor (VEGF). HIF is a potent stimulator of angiogenesis that coordinates the processes of neovascularization and angiogenesis by targeting VEGF and other pro-angiogenic factors (Zhou et al., 2019). In tumors, the inhibition of PHD2 in endothelial cells normalizes tumor vasculature by upregulating HIF, thereby sensitizing tumors to chemotherapy (Koyama et al., 2017). Therefore, upregulation of HIF might be an effective strategy for promoting angiogenesis and normalization of blood flow.

A study of diabetic rats reported that roxadustat promoted angiogenesis and wound healing by upregulating the HIF-1α/VEGF/VEGFR2 pathway (Zhu et al., 2019). A study of renal IRI in mice reported that roxadustat attenuated renal injury by enhancing renal angiogenesis and tissue repair due to its upregulation of HIF (Wu et al., 2021). A study of neonatal mice found that intraperitoneal roxadustat upregulated the expression of HIF-1α, VEGF, and endothelial NO synthase, promoted alveolar normalization and angiogenesis, and alleviated hyperoxia-induced lung injury (Huang et al., 2021). Another study of roxadustat reported that it significantly upregulated VEGF production in rats and bone marrow mesenchymal stem cells, and also promoted fracture healing (Chen et al., 2021). Two animal studies showed that VEGF promoted neurogenesis and also had antidepressant effects (Jin et al., 2002; Deyama et al., 2019). Li et al. (2020c) studied a rat model of depression and showed that roxadustat appeared to reverse depression-like behaviors in rats by activating HIF and its target genes (*EPO* and *VEGF*), and it also reduced memory impairment. Studies of preterm infants found that hyperoxia led to downregulation of HIF, and this may have led to attenuated retinal vessel growth and vascular occlusion, leading to retinopathy of prematurity (ROP) (Hartnett and Penn, 2012). There is also evidence that the activation of HIF by roxadustat prevented oxygen-induced retinopathy in animal models, suggesting its possible use for prevention of childhood blindness (Hoppe et al., 2016; Liu et al., 2016). Another study of ROP found that roxadustat stably increased the expression of glycolysis-related genes by inducing HIF-1α, which improved retinal metabolism and normalized angiogenesis (Hoppe et al., 2020). An elevated level of HIF-2α contributes to angiogenesis during retinopathy, and roxadustat provides a weak induction of HIF-2α in retinal cells, thus suggesting the safety of this drug for treatment of ROP (Hoppe et al., 2020). Zhou et al. (2019) examined a rat subcutaneous cavity model and showed that roxadustat promoted angiogenesis and maturation *in vitro* and *in vivo*, suggesting it may be useful during tissue transplantation.

On the other hand, hypoxia is also a key characteristic of the tumor microenvironment, and HIF overexpression occurs in a variety of cancers (Talks et al., 2000). Theoretically, long-term application of a HIF activator could increase the risk for tumorigenesis and diabetic retinopathy. However, no clinical studies of roxadustat have reported that it increased the systemic level of VEGF, increased tumorigenesis or metastasis, or adversely affected diabetic retinal neovascularization (Akizawa et al., 2020a). Considering that roxadustat is an oral medication that is given intermittently, it is likely that a low level of cellular HIF activation is insufficient to increase VEGF expression, but is sufficient to increase erythropoiesis. However, a case report of a patient treated in a phase III clinical trial of roxadustat reported the development of pulmonary arterial hypertension (PAH) (Cygulska et al., 2019). Although confirmation in additional patients is necessary, stabilization of HIF by roxadustat may possibly contribute to the pathophysiology of PAH.

**FIGURE 5:**
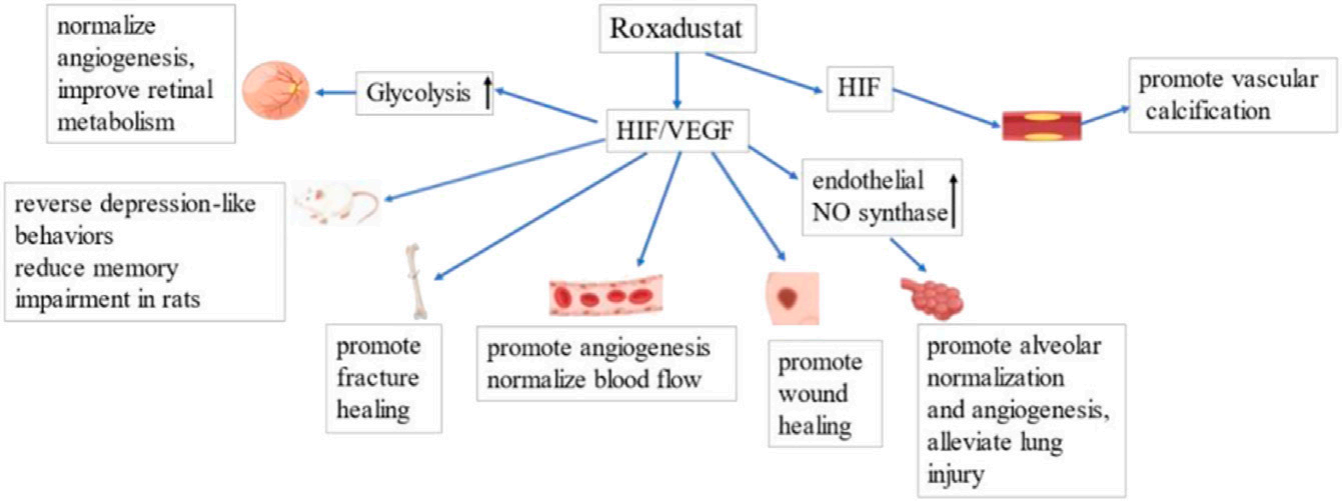
Roxadustat promotes angiogenesis by upregulating HIF/VEGF. Left to Right: Roxadustat promotes glycolysis, thereby improving retinal metabolism and normalizing blood flow. Roxadustat reverses depression-like behavior and reduces memory impairment in rats. Roxadustat promotes wound and fracture healing by promoting angiogenesis and normalization of blood flow. Roxadustat upregulates the expression of endothelial NO synthase, promotes alveolar normalization and angiogenesis, and thereby attenuates lung injury. Roxadustat may also lead to vascular calcification, increasing the risk of cardiovascular events.

Furthermore, HIF stabilization may promote wound healing and collateral vessel growth when there is cardiovascular injury (Flight, 2013). Some evidence suggests that HIF activation plays a cardioprotective role in myocardial IRI (Li et al., 2017), although prolonged HIF activation can promote vascular calcification. Vascular calcification is a common complication in CKD patients, and is closely related to their increased risk of cardiovascular complications (Zhu and Reiser, 2021). Mokas et al. (2016) found that HIF and phosphate acted synergistically to promote osteogenic transdifferentiation and calcification of vascular smooth muscle cells, and that roxadustat promoted vascular calcification. This finding suggests that roxadustat may possibly increase the risk for cardiovascular events in CKD patients. However, the potential role of elevated HIF in promoting vascular calcification remains unclear, and this topic should be examined when considering the possible adverse effects of HIF activation and roxadustat. Figure 5 summarizes the mechanisms by which roxadustat promotes angiogenesis and reduces vascular abnormalities in numerous diseases.

## Conclusion

Roxadustat is a HIF activator that is approved for the clinical treatment of renal anemia in China, the European Union, Japan, South Korea, and Chile. Numerous clinical studies showed that it is safe and effective in maintaining target Hb levels in CKD patients, and it is not significantly affected by inflammatory status. Thus roxadustat, as a new HIF-PHI, is an encouraging new option for the treatment of patients with CKD. In addition to the treatment of anemia in these patients, roxadustat also has potential for reducing tissue and organ fibrosis and inflammation, correcting metabolic disorders, reducing oxidative stress, improving mitochondrial function, and normalizing angiogenesis. Thus, roxadustat may provide benefits beyond reducing anemia. However, recent research has found that roxadustat, like all medications, must be used cautiously because of certain common adverse reactions. In particular, it has the potential to increase the risk of pulmonary hypertension and vascular calcification, and aggravate inflammatory infections. Although roxadustat appeared to cause no obvious off-target effects in clinical trials or clinical applications so far, HIF targets many different genes and has a wide range of effects. It is therefore necessary to strictly control the extent and duration of HIF activation, and to carefully consider the dose and schedule of roxadustat to avoid unexpected side effects caused by the pleiotropic effects of HIF activation.
